# Adenosine-Functionalized Biodegradable PLA-b-PEG Nanoparticles Ameliorate Osteoarthritis in Rats

**DOI:** 10.1038/s41598-019-43834-y

**Published:** 2019-05-15

**Authors:** Xiuling Liu, Carmen Corciulo, Stephanie Arabagian, Abraham Ulman, Bruce N. Cronstein

**Affiliations:** 1 Department of Chemical and Biomolecular Science and Engineering, NYU Tandon School of Engineering, 6 Metrotech Center, New York, NY 11201 USA; 2 Department of Medicine, NYU School of Medicine, 550 First Avenue, New York, NY 10016 USA

**Keywords:** Acute inflammatory arthritis, Drug delivery

## Abstract

Short biologic half-lives limit the therapeutic utility of many small molecules. One approach to extending the half-life of pharmacologically active small molecules is conjugation to less degradable nanoparticles; here we report the synthesis and activity of six targeted polymeric (PEG-*b*-PLA) nanoparticles for use as adenosine receptor agonists. Using click chemistry, PLA-*b*-PEG400-N_3_ and PLA-*b*-PEG2000 block copolymers were bound to adenosine at the 3′,4′-OH, 5′-OH, and 6-NH_2_ positions with an acetylene group. Activity of the conjugates as adenosine receptor ligands was tested by their capacity to stimulate cAMP increases in RAW264.7 murine macrophage cells. Only adenosine-conjugated nanoparticles (A-3′,4′-OH-TPN2), in which PEG2000 was bound to adenosine on the 3′,4′ hydroxyl groups, stimulated cAMP increases and these increases were blocked by selective antagonists of both adenosine A2A and A2B receptors, consistent with ligation of these receptors. Adenosine nanoparticles were tested *in vivo* in a rat model of post-traumatic osteoarthritis; intra-articular injection of adenosine nanoparticles prevented the development of osteoarthritis in this model. These studies suggest that attachment of adenosine to biodegradable nanoparticles provides a novel approach to achieving prolonged therapeutic effects.

## Introduction

Biodegradable nanoparticles (NPs) have gained increasing interest for their ability to provide a viable carrier for delivery of vaccines, genes, drugs and other biomolecules. Their enhanced biocompatibility and prolonged release profiles make them useful for a variety of medical applications^1^. Biodegradable NPs improve solubility of hydrophobic drugs, increase local concentration, provide longer clearance time, increase probability of interactions (e.g. when activation of a receptor is critical), and generally have low toxicity. For example, it has been observed that to avoid clearance by the reticuloendothelial system (RES), the addition of polyethylene glycol (PEG) on the surface of NPs is required^2^. As a consequence of surface PEG molecules, higher maximum tolerated doses (MTD) of nanoparticles (NPs) are realized^3^.

Osteoarthritis (OA) is the most common type of arthritis affecting as many as 29 million people in the United States alone. One in every two people will likely be affected by osteoarthritis. OA is a degenerative joint disorder in which the articular cartilage is destroyed and chondrocytes, the cells that synthesize and maintain cartilage, play a central role in cartilage destruction. Age, injury and inflammation reduce the capacity of chondrocytes to synthesize and maintain ATP, a molecule which is important for maintaining chondrocyte and cartilage homeostasis. The pathogenesis of osteoarthritis involves low grade inflammation, destruction of articular cartilage and reactive overgrowth of bone in the affected joints. At the present time, therapy is, for the most part, palliative including use of nonsteroidal anti-inflammatory drugs (e.g. ibuprofen^4,5^), narcotic analgesics^6^, exercise, acupuncture^7,8^, and injections of anti-inflammatory agents (e.g. glucocorticoids^9^) or other substances (hyaluronic acid^10,11^) into the joint. Thus, short of total joint replacement, there are few long-term effective therapies available. Notice, that although currently available injectable agents (corticosteroids, hyaluronate) provide symptomatic relief, none of these agents are restorative. Therefore, improved and more effective treatments of osteoarthritis are required.

Recently, Corciulo and coworkers have shown that intracellular and extracellular levels of ATP fall after treatment of mouse chondrocytes and rat tibia explants with IL-1β, an inflammatory mediator that participates in OA pathogenesis^12^. In the extracellular space ATP is hydrolyzed to adenosine which is a ligand for a family of cell surface G protein coupled receptors (A1R, A2AR, A2BR, A3R). Mice deficient in the A2A adenosine receptor (A2AR) or the ecto-enzyme that mediates the conversion of AMP to adenosine (ecto-5′nucleotidase) develop spontaneous OA^12^. Adenosine itself is extremely short-lived in biologic fluids; in whole blood adenosine has a half-life of 1–4 seconds and likely has a similar short half-life in the joint^13^. In a rat model of post-traumatic OA the intra-articular injection of liposomal suspensions containing adenosine prevents development of OA^12^. Based on these results these authors formulated the hypothesis that extracellular adenosine is an important homeostatic mechanism for chondrocytes and loss of adenosine-A2AR-mediated chondrocyte homeostasis contributes to OA development. It follows that adenosine A2A receptors might be an excellent target for the treatment or prevention of OA.

Whereas liposomes are excellent for microencapsulation and drug delivery, once open and their cargo is released, the clearance time of the drug does not significantly increase relative to its application as a molecular formulation. Hence, the relative advantage of the liposomes is their prolonged release process. Therefore, the next logical step is the attachment of the molecule under study (adenosine) to a carrier by a chemical bond. For these studies we have selected poly(lactic acid)-poly(ethylene glycol) (PLA-PEG) nanoparticles (NPs). While the majority of PLA-PEG NPs have been used for encapsulation of bioactive molecules, surface functionalized PLA-PEG NPs are ideal when activation of the receptor is required^14,15^. Adenosine A2A receptor inhibits osteoclast differentiation and activates osteoblasts, stimulates chondrocytes and suppresses inflammation, among many other effects^16–18^. Agents that target A2AR effectively inhibit inflammatory osteolysis in inflamed bone^17^. Thus, an agent that targets A2AR could be a new therapeutic that promotes bone regeneration and increases bone volume.

One of the concerns in using PLA-PEG nanoparticles is their hydrolysis to lactic acid. However, it has been well established that the damage done during OA is mediated as a result of changes in the chondrocytes, not the acid milieu. The chondrocytes have to be alive to mediate OA damage to the cartilage^18^. Here we report on the first preparation of adenosine-functionalized PLA-PEG NPs, and their use for treatment in a rat model of post-traumatic osteoarthritis. Our hypothesis is that such NPs will be highly effective, since given the number of surface functionalized adenosine molecules, one NP can potentially activate more than one receptor.

## Results and Discussion

### Synthesis of adenosine-functionalized pla-b-peg copolymers

**Figure 1:**
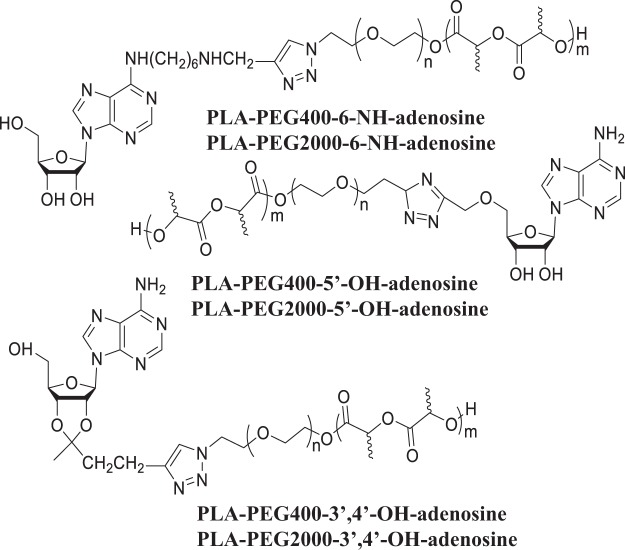
Adenosine-terminated PLA-PEG block copolymers.

Adenosine has three potential sites for polymer attachment –the 3′,4′-OH groups, the 5′-OH group, and the 6-NH_2_ group. Our hypothesis has been that these different attachments should result in adenosine, having free NH_2_ and/or OH groups with different spacing, hence with different H-bonding schemes (molecular recognition), different H-bonding strength, and thus different interaction energies with the receptor, which might result in different bioactivities. Figure 1 shows the six adenosine-terminated PLA-PEG block copolymers prepared in this study. Block copolymers had been prepared using both PEG400 and PEG2000.

While PLA-PEG biodegradable nanoparticles have been made before, the attachments of adenosine using the 3′,4′-OH, 5′-OH, and 6-NH_2_ groups have raised significant synthetic challenges, and has required the development of new strategies for functionalized adenosine molecules. The overall strategy for the synthesis of adenosine functionalized biodegradable nanoparticles involves four parts: 1. Synthesis of poly[lactic acid]-poly[ethylene glycol] (PLA-PEG) block copolymer terminated with an azide group (PLA-b-PEG-N_3_); 2. Synthesis of adenosine functionalized with an acetylene group (–C≡C–H); 3. Connection of the adenosine to the block copolymer using click chemistry between the acetylene and the azide; 4. Preparation of nanoparticle from the adenosine-functionalized block copolymer. NPs for control experiments do not carry adenosine, and the PEG is terminated with a methyl group. All syntheses described here were the same for PEG400 and PEG2000.

### Synthesis of PLA-b-PEG-N_3_

**Figure 2:**
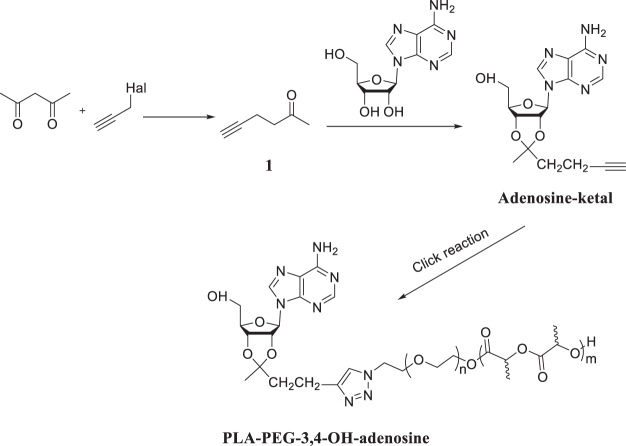
Adenosine-terminated PLA-PEG block copolymers.

The synthesis of HO-PEG-N_3_ was carried out as described by Mahou and Wandrey^19^ (Fig. 2). Ring opening polymerization of D,L-lactide was performed using HO-PEG-N_3_ in dry toluene, with Sn(Oct)_2_ as the catalyst. The product was purified by precipitation from dichloromethane solution using diethyl ether.

### Synthesis of PLA-PEG-3′,4′-OH-adenosine

**Figure 3:**
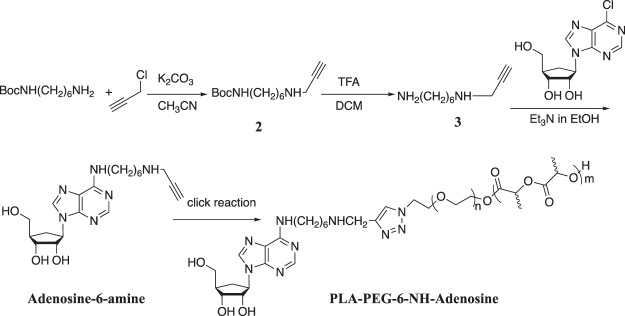
Synthesis of PLA-b-PEG400-6-NH-adenosine, and PLA-b-PEG2000-6-NH-adenosine.

Figure 2 shows the synthetic strategy. An attachment through the 3′,4′-OH groups is accomplished by reacting the with a ketone, forming a 5-membered ketal ring. The attachment to the azide-terminated PLA-PEG block copolymer, using click chemistry, requires an acetylene group, hence we first prepared 5-Hexyn-2-one (1 in Fig. 3) using a method reported by Görl and Alt^20^. 2,4-Pentanedione and propargyl chloride were allowed to react in ethyl alcohol containing anhydrous potassium carbonate resulting in the desired compound. This ketone was then reacted with adenosine in anhydrous DMF, using triethyl orthoformate as water scavenger, and HCl in dioxane as a catalyst^21^, yielding the desired ketal, which could be purified using chromatography. The reaction of alkynated adenosine with PLA-b-PEG-N_3_ was carried out in anhydrous DMF, using CuBr and the catalyst and N,N,N′,N′′,N′′-pentamethyl-diethylenetriamine (PMDETA) as the base (Copper(I)-catalyzed azide-alkyne cycloaddition –“click chemistry”)^22^. The product was purified by successive precipitations from THF using water, and was freeze-dried to yield a white powder.

### Synthesis of PLA-b-PEG-6-NH-adenosine

Figure 3 presents the synthetic strategy. The attachment of the PLA-b-PEG to adenosine was accomplish by first reacting an acetylene-terminated primary hexamethylene diamine with 6-chloro-9-(β-D-ribofuranosyl)-purine. A reaction of mono Boc-protected hexamethylenediamine with propargyl chloride in anhydrous acetonitrile using K_2_CO_3_ as acid scavenger, resulted in tert-butyl(6-(pro-2-yn-1-ylamino)hexyl)carbamate in 68% yield after column chromatography on a basic alumina (Dichloromethane: MeOH = 50:1). The removal of the Boc protecting group was accomplished in 96% yield using trifluoroacetic acid in dichloromethane. The resulting free amine was reacted with 6-chloro-9-(β-D-ribofuranosyl)purine in ethanol, using trimethylamine as the acid scavenger. Purification using silica gel chromatography (CH_3_OH:Dichloromethane, 1:10) provided the desired product in 50% yield. A click reaction with the azide-terminated PLA-b-PEG - as described above –provides the PLA-b-PEG-6-NH-adenosine.

### Synthesis of PLA-b-PEG-5′-OH-adenosine

**Figure 4:**
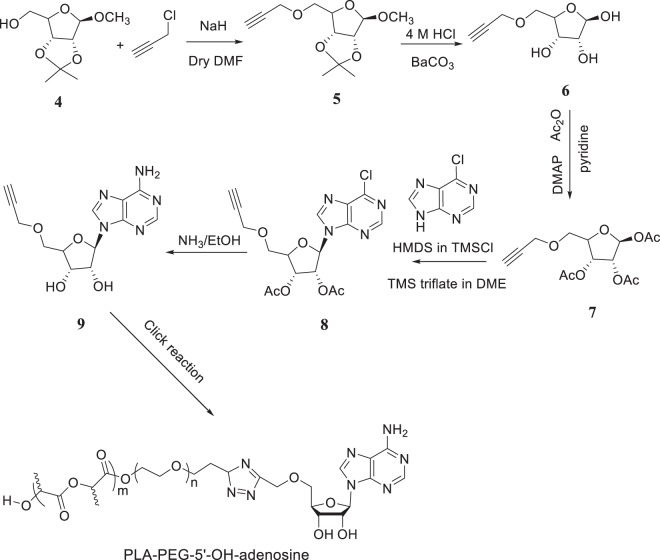
Synthesis of PLA-b-PEG400-5′-OH-adenosine, and PLA-b-PEG200-5′-OH-adenosine.

The synthetic strategy for the synthesis of PLA-b-PEG-5′-OH-adenosine is presented in Fig. 4. This synthesis is more involved since the purine and the furanose parts of the adenosine must be connected. Methyl 5-O-propargyl-2,3-O-isopropylidene-β-D-ribofuranose was prepared first deprotonating 2,3-O-isopropylidene-β-D- ribofuranoside by NaH in dry DMF. The alkoxide was allowed to react with propargyl chloride, providing compound 5 in Fig. 4. The methyl group was removed using HCl to yield compound 6, which was then actylated using acetic anhydride in dry pyridine with DMAP as acid scavenger, providing compound 7. Next, 6-Chloropurine was treated with 1,1,1,

3,3,3-hexamethyldisilazane and chlorotrimethylsilane, and the silylated compound, which was used without further purification, in Vorbruggen coupling with compound 7 to yield compound 8. Finally, compound 8 was allowed to react with ammonia in ethanol to yield the desired adenosine derivative. A click reaction with the azide-terminated PLA-b-PEG - as described above –provides the PLA-b-PEG-5′-OH-adenosine.

### Synthesis of adenosine-functionalized nanoparticles

**Figure 5:**
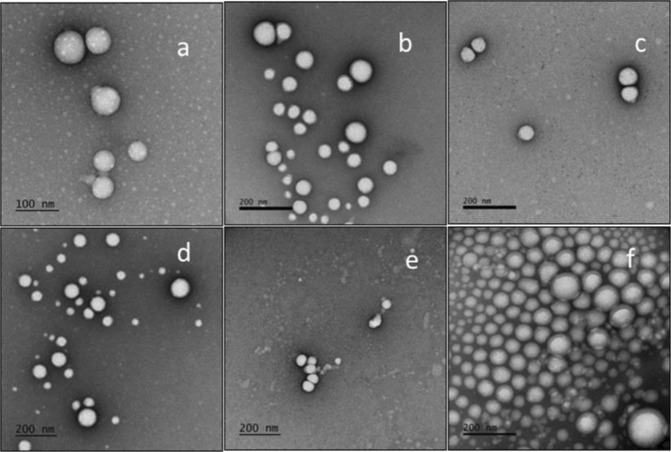
The shape of adenosine-functionalized nanoparticles. Transmission electron microscopy images for six nanoparticles. (**a**) PLA-PEG400-3′,4′-OH-adenosine; (**b**) PLA-PEG400-6-NH-adenosine; (**c**) PLA-PEG400-5′-OH-adenosine; (**d**) PLA-PEG2000-3,4-OH-adenosine; (**e**) PLA-PEG2000-6-NH-adenosine; (**f**) PLA-PEG2000-5-OH-adenosine.

Nanoparticles were made by dissolving the polymer in ethyl acetate and adding it to an aqueous Pluronic F68 solution. An emulsion was formed using a vortex shaker, which was then ultrasonicated, and the organic solvent was removed under reduced pressure. The resulting nanoparticle suspension was ultracentrifuged, and the resulting pellet was re-suspended in pH = 7.4 PBS. The nanoparticles were filtered through a 1 µm glass filter fist and then 0.45 µm and stored at 4 °C until use. The following nanoparticles were prepared: PLA-PEG400-3′,4′-OH-adenosine, PLA-PEG2000-3′,4′-OH-adenosine, PLA-PEG400-5′-OH-adenosine, PEG2000-5′-OH-adenosine, PEG400-NH-adenosine, and PEG2000-NH-adenosine. Nanoparticle size and size distribution (S1-7) were determined by dynamic light scattering (Table 1). Transmission electron microscopy studies of the six adenosine attached copolymer nanoparticles showed an exclusively spherical morphology in Fig. 5. Because the nanoparticles aggregate when the temperature increases and TEM data collection takes three hours, during which time the temperature of the nanoparticles increases from 4 °C (their storage temperature) to room temperature, there is some variability in the apparent size. Moreover, nanoparticles tend to aggregate at high concentrations. Nonetheless, the TEM images in Fig. 5 show both single and aggregated nanoparticle clusters.

**Table 1:** Diameter of nanoparticles (light scattering measurements).

	Diameter Size (nm)	PDI
PLA-PEG400-3′,4′-OH-adenosine	131	0.156
PLA-PEG400-6-NH-adenosine	141	0.133
PLA-PEG400-5′-OH-adenosine	137	0.145
MeOPEG550-PLA(Control)	133	0.091
PLA-PEG2000-3,4-OH-adenosine	129	0.126
PLA-PEG2000-6-NH-adenosine	144	0.159
PLA-PEG2000-5-OH-adenosine	140	0.181

### Stimulation of adenosine receptors in RAW264.7 cells

**Figure 6:**
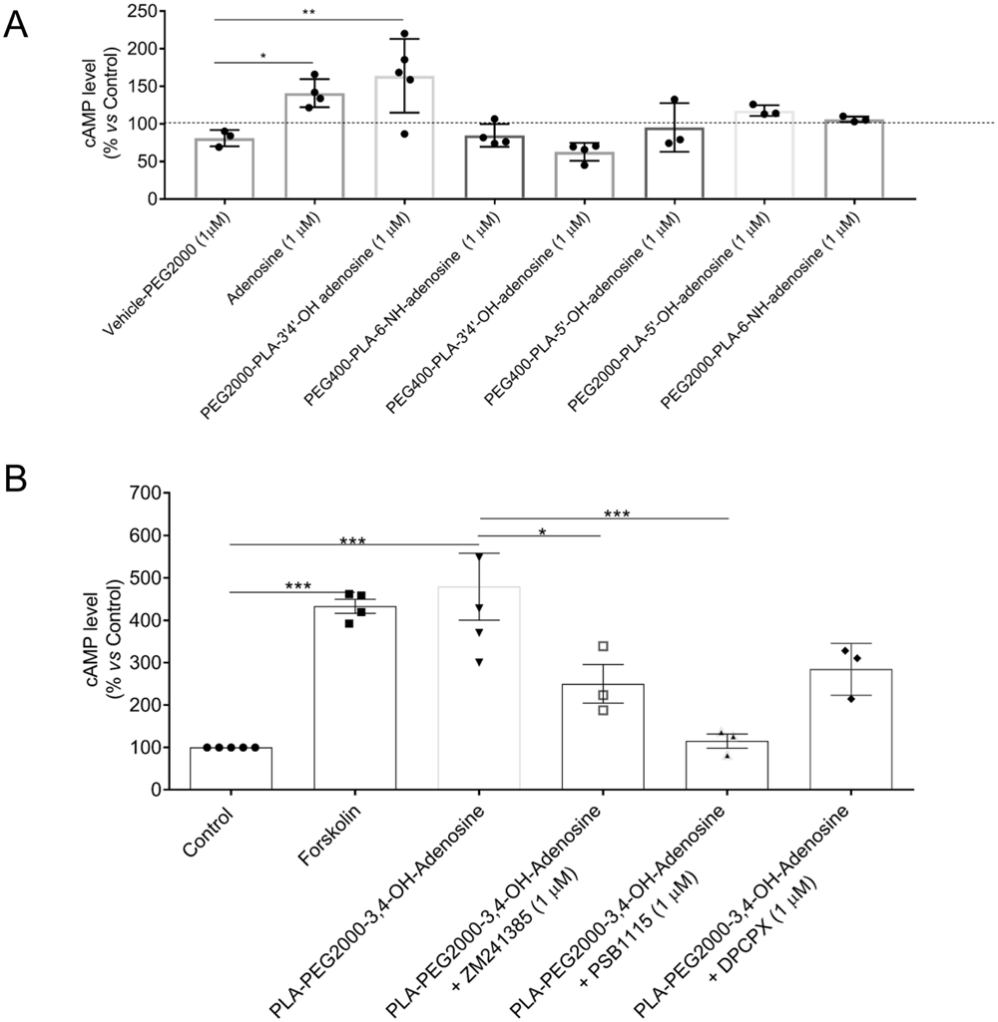
Adenosine and Adenosine functionalized nanoparticles stimulate cAMP accumulation. (**A**) Adenosine and PLA-PEG2000-3,4-OH-adenosine (PEG2000-Ado) but not PEG400-Ado or PEG2000-PLA-5′-OH-adenosine or PEG2000-PLA-6-NH-adenosine particles stimulate a significant increase in cellular content of cAMP as compared to vehicle in the RAW 264.7 cell line. (**B**) Adenosine A2A and A2B antagonists reverse the effect of adenosine functionalized nanoparticles on cAMP accumulation. Co-treatment with A2AR antagonist ZM241385, the A2BR antagonist PSB1115 and the poorly selective A1R/A2R antagonist DPCPX significantly reduced the PEG2000-Ado effect on cAMP production. Data are expressed as % control cAMP content and each data point shown represents results of experiments carried out on a single day in duplicate. (*p < *0.05*; **p < *0.01*; *n* = *3*–*5*).

To determine whether the adenosine-conjugated nanoparticles stimulate adenosine receptors in RAW264.7 cells, we tested the effect of NPs alone (control), adenosine, and the six types of adenosine-conjugated NPs on cAMP accumulation. As shown in Fig. 6A, following incubation of RAW264.7 cells incubation with adenosine alone stimulated a 50% increase in cellular cAMP and only the PEG2000 nanoparticles conjugated to adenosine at the ribose 3′,4′-OH groups stimulated a similar and significant increase in cAMP accumulation. None of the adenosine conjugated to PLA-PEG400 nanoparticles had any effect on cAMP accumulation. Similarly, the 5′-OH- and amine-conjugated PLA-PEG2000 nanoparticles had no significant effect on cAMP content.

To understand the pharmacologic mechanism by which the adenosine-conjugated particles acted on cells we determined whether selective adenosine A2A and A2B receptor antagonists blocked the effect of the particles on cAMP accumulation in primary murine chondrocytes. To determine maximal cAMP increases in these cells we incubated the cells with forskolin, an agent which bypasses cell surface receptors to stimulate accumulation of cAMP in the primary cells (Fig. 6B). Forskolin induced a 4-fold increase in cAMP which was similar to that induced by the PLA-PEG2000-3′,4′-OH-adenosine particles. We found that the adenosine-conjugated nanoparticles stimulated cAMP accumulation via both A2A and A2B adenosine receptors since co-incubation with selective antagonists for either A2A receptor (ZM241385), or A2B receptor (PSB1115) diminished the effect of the adenosine nanoparticles on cAMP accumulation (Fig. 6B).

**Figure 7:**
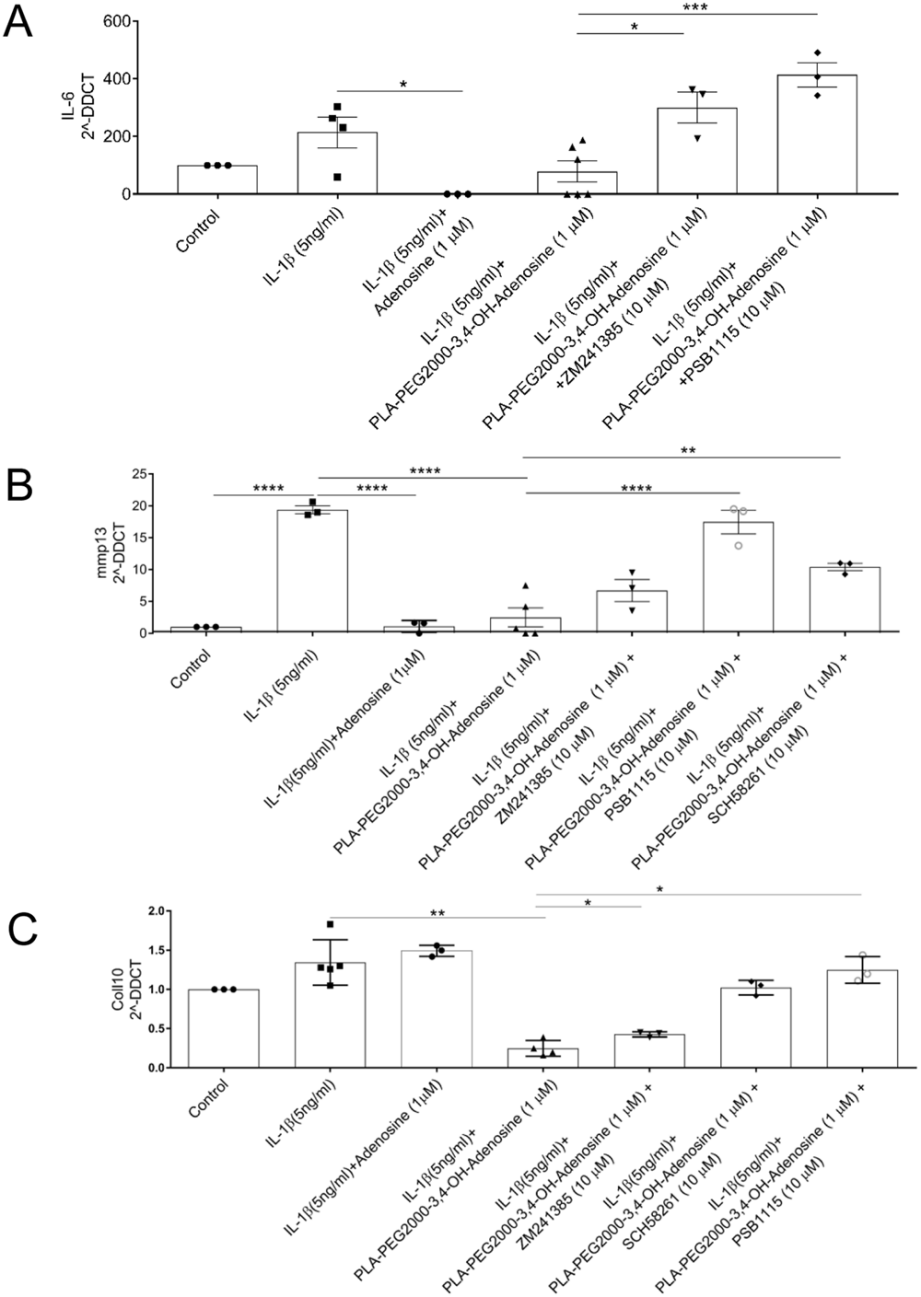
Adenosine functionalized PLA nanoparticles regulate expression of inflammatory mediators and markers. Chondrocytes were incubated with IL-1b, nanoparticles and adenosine A2A receptor antagonists (ZM241385 and SCH58261) or an A2B antagonist (PSB1115), as described. RNA was isolated and subject to reverse transcription and real time-PCR, as described and levels of specific mRNA were calculated and normalized to LDH. The functionalized nanoparticles diminished message for: (**A**) IL-6; (**B**). MMP-13, and; (**C**). Col10a1. Each point represents a separate determination. (*p < *0.05*; **p < *0.01*; ***p < *0.001*; *n* = *3*–*5*).

**Figure 8:**
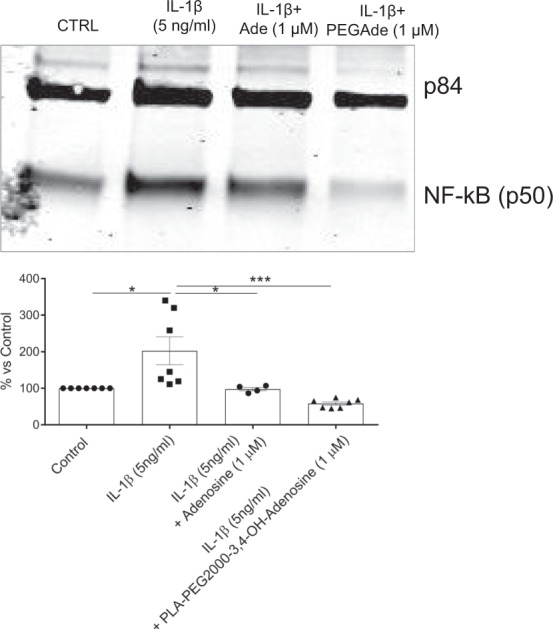
PEG2000-Ado NPs and adenosine decrease IL-1β induced NF-kB nuclear translocation in primary murine chondrocytes. Primary murine chondrocytes were cultured in the absence (Control) or presence of IL-1β and either adenosine or adenosine conjugated nanoparticles for 24 hours at 37 °C. Nuclear fractions were isolated, as described, and proteins isolated. After separation of proteins by electrophoresis through 10% polyacrylamide gels the proteins were then transferred to a nitrocellulose membrane, as described. Nuclear NFkB and P84 (a control nuclear protein) were detected following decoration with appropriate antibodies and quantitated by densitometry of bands on Western Blot. Data are expressed as % control levels. Each point represents results from an individual experiment (*p < *0*.*05*; ***p < *0*.*001*; *n* = *4*–*7*).

To further pinpoint which adenosine receptors are activated by the adenosine-conjugated nanoparticles and their effect on chondrocyte function we tested the effects of the particles on expression of message for inflammatory mediators in primary murine chondrocytes. We observed that adenosine-conjugated nanoparticles diminished the IL-1β-stimulated expression of IL-6, MMP13 and Collagen 10, an effect that was partially reversed by A2AR (ZM241385 and SCH58261) and A2BR (PSB1115) antagonists (Fig. 7A). In contrast, as shown in Fig. 7B,C, the A2AR antagonists only partially reversed the effect of the adenosine-conjugated nanoparticles on IL-1β-stimulated MMP13 and Collagen-10 expression whereas the A2BR antagonist completely reversed this effect. These findings suggest that, at the concentrations used, A2AR antagonists are not as potent as the A2BR antagonist for reversing the effect of the conjugated nanoparticles on cells. Both the adenosine-conjugated nanoparticles and adenosine alone diminished IL-1β stimulated activation of NF-kb, a central intracellular signal for inflammation (Fig. 8).

Taken together the results of the *in vitro* studies clearly demonstrate that the adenosine-functionalized particles bind to and activate adenosine A2A and A2B receptors on murine cells as well as adenosine. Moreover, like adenosine the particles are not selective for A2A or A2B receptors. Because prior experiments had demonstrated that PLA polymers are degradable *in vivo* we next investigated the effect of these nanoparticles in the prevention of osteoarthritis in a rat model^23,24^.

### Intra-articular injection of nanoparticles prevents progression of osteoarthritis in a rat post-traumatic osteoarthritis model

**Figure 9:**
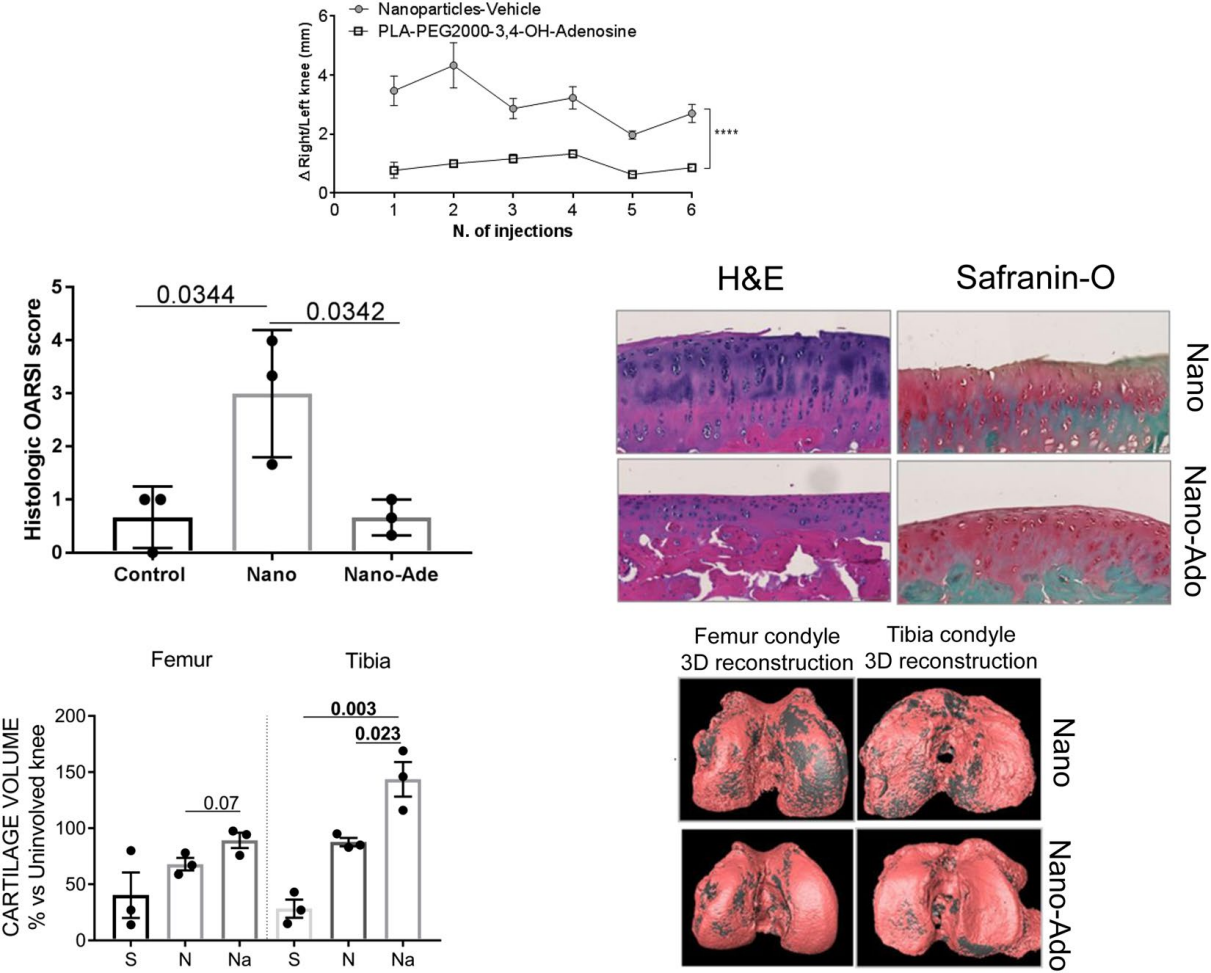
Adenosine-conjugated NPs preserve articular cartilage in a rat model of post traumatic osteoarthritis. Intra-articular injection of adenosine-conjugated NPs diminishes knee swelling (**A**). Histologic analysis reveal cartilage protection with less fibrillation, proteoglycan loss (red surface in the Safranin-O stained slides) and decrease of OARSI score in Nano-Ado treated rats compared to the rats treated with the vehicle (**B**). Moreover, the reconstruction of the µCT data reveals a reduction of the cartilage surface damage (**C**); cartilage is pink and the bone below is grey). The total volume of the cartilage in the OA knee compare to the uninvolved knee is 68% in the vehicle group and 89% of the Nano-Ado group (****p < 0.0001; n = 3 for each group).

Because we found that the adenosine-conjugated PEG2000 NPs acted at adenosine A2AR and A2BR, we next determined whether these NPs could ameliorate damage in a rat post-traumatic osteoarthritis model. As shown in Fig. 8, intra-articular injection of the adenosine-conjugated nanoparticles diminished swelling in affected knees whereas unconjugated nanoparticles had no effect on knee swelling (Fig. 9A). In rats treated with adenosine conjugated-NP there was markedly reduced fibrillation of the cartilage surface (H&E staining) and less proteoglycan loss (Safranin-O staining) resulting in a significantly decreased OARSI score as compared to the unconjugated nanoparticles (Fig. 9B). More importantly, intra-articular injection of the knees with adenosine-conjugated nanoparticles prevented loss of cartilage (the pink material in these reconstructed microCT images, Fig. 9C), as compared to the unconjugated nanoparticles. These results provide further evidence that intraarticular injection of an adenosine receptor agonist is useful for the treatment of osteoarthritis.

Corciulo and colleagues recently reported that endogenously generated adenosine plays a critical role in maintaining chondrocyte and cartilage homeostasis^12^. Because injections of adenosine alone, which has an extremely short half-life (1–4 seconds) in biological fluids^13^, does not affect the course of osteoarthritis^12^ we have sought to develop alternative approaches to delivery of adenosine or adenosine receptor agonists with a prolonged duration of action to osteoarthritic cartilage we developed adenosine-conjugated nanoparticles. Here we report that intraarticular injection of adenosine-conjugated nanoparticles, which act as adenosine receptor agonists, but not unconjugated nanoparticles prevents progression of post-traumatic osteoarthritis in a rodent model. These nanoparticles may provide an appropriate therapeutic for the treatment of osteoarthritis.

## Conclusion

We report the synthesis and activity of three adenosine-functionalized PLA-b-PEG biodegradable NPs. Using click chemistry, PLA-b-PEG-N3 block copolymers were connected to adenosine at the 3′,4′-OH, 5′-OH, and 6-NH2 positions with an acetylene group. Of the three different adenosine-functionalized NPs, one, in which the copolymer was bound to the adenosine on the 3′,4′-hydroxyl groups, induced cellular increases in cAMP, in an adenosine A2A and A2B receptor-dependent fashion. These NPs were then tested in a rat model of post-traumatic osteoarthritis and were found to effectively block the development of osteoarthritis in this model. We conclude that adenosine-functionalized PLA-b-PEG particles are a novel approach to developing long lasting therapies for local treatment of such conditions as osteoarthritis.

## Material and Methods

### Material

Poly(ethylene glycol) (PEG400), poly(ethylene glycol) (PEG2000), propargyl chloride, methyltrichlorosilane, stannous octoate, adenosine, p-toluene sulfonyl chloride, sodium azide, 2,4-pentanedione, anhydrous potassium carbonate, triethyl orthoformate, sodium bicarbonate, trifluoroacetic acid, triethyl amine, sodium hydride, copper bromide, barium carbonate, concentrated hydrochloric acid, 4-dimethylaminopyridine, acetic anhydride, 1,1,1,3,3,3-hexamethyldisilazane, chlorotrimethylsilane, TMS triflate, 4% NH_3_/EtOH, Pluronic Methoxypoly(ethylene glycol), potassium iodide. N-boc-1,6-diaminohexane, N,N,N,N,N″ pentamethyldiethylenetriamine, 6-chloro-9-(β-D-ribofuranosyl)purine, silver oxide, 6-chloro-9-(β-D-ribofuranosyl)purine and 6-Chloro-7H-purine, Methyl 2,3-O-isopropylidene-β-D-ribofuranoside were purchased from commercial sources and used as received. D,L-Lactide was recrystallized from toluene then dried overnight before used. Phosphate buffered saline (PBS) was purchased from Boston BioProduct. Glass filter were purchased from Sterlitech Corporation and 0.45 µm filter was purchased from Pall Corporation. All common solvents were used as received without further distillation. Adenosine receptor antagonists 8-Cyclopentyl-1,3-dipropylxanthine (DPCPX, A1/A2 receptor antagonist), 4-(-2-[7-amino-2-{2-furyl}{1,2,4}triazolo{2,3-a} {1,3,5}triazin-5-yl-amino]ethyl)phenol (ZM241385, A2A receptor antagonist), 7-(2-phenylethyl)-5-amino-2-(2-furyl)-pyrazolo-[4,3-e]-1,2,4-triazolo[1,5-c]pyrimidine (SCH58261, A2A receptor antagonist), 1-Propyl-8-(4-sulfophenyl)xanthine (PSB1115, A2B receptor antagonist) were purchased from SigmaAldrich.

### Use of experimental animals

These experiments were reviewed and approved by the NYU School of Medicine Institutional Animal Care and Use Committee. All procedures were performed in accordance with the guidelines set forth by the Institutional Animal Care and Use Committee.

### Synthetic procedures

#### Synthesis of a-methoxyl-ω-hydroxyl PEG

Glassware was silanized by rinsing with a 5% methyltrichlorosilane solution in toluene, then with acetone and finally dried overnight at 130 °C^22^. To a mixture of MeO-PEG550 (293 mg, 0.12 mmol) and D,L-lactide(7.01 g, 48.62 mmol) was added, under dry condition, a solution of Sn(Oct)_2_ (2 drops) in anhydrous toluene (11.2 ml). The reaction mixture was degassed by vacuum and filled by nitrogen and then stirred in a preheated oil bath at 110 °C for 24 h. Toluene was removed under reduced pressure, and the obtained product was dissolved into a minimum volume of dichloromethane and subsequently precipitated in ethyl ether. The precipitate was then dissolved into a minimum amount of THF, further precipitated in water, and subsequently was dried over vacuum overnight. (MeO-PEG550, M_n,NMR_ = 18725 g/mol). ^1^H NMR (500 MHz, CDCl_3_): δ = 5.21–5.15 (m, 300 H, CH PLA), 3.73–3.80 (m, 4 H), 3.63 (m, 58 H, CH_2_ PEG), 3.38 (s, 3 H), 1.58 (m, 974 H, CH_3_ PLA) ppm.

#### Synthesis of PLA-β-PEG-N_3_

A typical synthesis is as follows. Glassware was silanized by rinsing with a 5% methyltrichlorosilane solution in toluene, then with acetone and finally dried overnight at 130 °C^25^. To a mixture of HO-PEG400-N_3_ or (HO-PEG2000-N_3_) (0.12 mmol) and D,L-lactide(7.01 g, 48.62 mmol) was added, under dry condition, a solution of Sn(Oct)_2_ (2 drops) in anhydrous toluene(11.2 ml). The reaction mixture was degassed by vacuum and filled by nitrogen and then stirred in a preheated oil bath at 110 °C for 24 h. Toluene was removed under reduced pressure, and the obtained product was dissolved into a minimum volume of dichloromethane and subsequently precipitated in ethyl ether. The precipitate was then dissolved into a minimum amount of THF, further precipitated in water, and subsequently was dried over vacuum overnight. (PLA-PEG400-N_3_, M_n,NMR_ = 18975 g/mol. PLA-PEG2000-N_3_, M_n,NMR_ = 21890 g/mol) PLA-PEG2000-N_3_, ^1^H NMR (500 MHz, CDCl_3_): δ = 5.21–5.15 (m, 30 H, CH PLA), 3.76 (m, 2 H, CH_2_ PEG), 3.68 (m, 2 H, PEG), 3.66 (m, 14 H, PEG), 1.58 (m, 974 H, CH_3_ PLA). IR (Nujol): υ = 2944 (w), 2353 (w), 1750 (s), 1182 (s), 1080 cm^−1^ (s).

#### Synthesis of 5-hexyn-2-one (Compound 1 Fig. 2)

2,4-Pentanedione (5 g, 5 ml, 50 mmol), anhydrous potassium carbonate (7.5 g, 55 mol), and propargyl chloride (3.5 g, 3.5 ml, 48 mmol) were dissolved in 25 ml of ethanol^19^. The reaction mixture was stirred under reflux for 24 h. After cooling to room temperature, 15 ml of water were added. The mixture was then extracted with diethyl ether, and the organic phase was washed with brine and dried over sodium sulfate. Removal of the solvent and subsequent vacuum distillation yielded 5-hexyn-2-one as colorless liquid in 48% yield.

#### Synthesis of adenosine-ketal

5-hexyn-2-one (0.5 g, 5.21 mmol), adenosine (1.4 g, 5.24 mmol) and triethyl orthoformate (0.776 g, 5.24 mmol) were dissolved in 18 ml of DMF^20^. Then the 4 M HCl in 1,4-dioxane (4.08 ml) was added to the mixture. The reaction mixture was stirred under room temperature overnight. The reaction mixture was poured into diethyl ether (250 ml). Then the oil residue was dissolved in chloroform (100 ml) and the organic phase was washed with sodium bicarbonate solution (2%) for one time and then water for three times, then dried over anhydrous MgSO_4_. The solvent was removed via rotary evaporator. The crude product was purified through chromatography (CHCl_3_:CH_3_OH = 10:1). ^1^H NMR (CDCl_3_) δ 8.32 (s, 1 H), 7.85 (s, 1 H), 6.49 (d, *J* = 10 Hz, 1 H), 5.92 (d, *J* = 5 Hz, 2 H), 5.85 (s, NH_2_, 2 H), 5.24 (d, *J* = 4 Hz, 1 H), 5.23 (d, *J* = 4 Hz, 1 H), 4.54 (s, 1 H), 3.99 (d, *J* = 13 Hz, 1 H), 3.82 (d, *J* = 13 Hz, 1 H), 2.47 (m, 2 H), 2.15 (t, 2 H), 2.03 (s, CH, 1 H), 1.36 (s, 3 H) ppm. IR (Nujol): υ = 3628 (w), 3312 (s), 3250 (m), 3098 (m), 2703 (m), 2100 (w), 1687 (s), 1608 cm^−1^ (s)

#### Synthesis of tert-Butyl(6-(pro-2-yn-1-ylamino)hexyl)carbamate (Compound 2, Fig. 3)

Propargyl chloride (344.4 mg, 4.6 mmol, 1 equiv.), Boc-protected amine (1.00 g, 4.62 mmol, 1 equiv.) and anhydrous K_2_CO_3_ were in acetonitrile. The reaction was stirred at room temperature for 16 h. Then, 100 ml of water and 100 ml of diethyl ether were added. The organic phase was separated and the aqueous phase extracted with diethyl ether (2 × 50.0 ml). The solvent was removed under reduced pressure. The crude product was purified by column chromatography on a basic alumina (CH_2_Cl_2_:CH_3_OH = 50:1). After drying on high-vacuum the product was obtained as a yellow oil (0.7991 g, 68%) yield. ^1^H NMR (CDCl_3_) δ 4.5 (s, 1 H, NH), 3.45 (s, 2 H), 3.14 (d, 2 H), 2.72 (m, 2 H), 2.23 (s, 1 H), 1.5 (s, 8 H), 1.46 (s, 9 H) ppm.

#### Synthesis of 6-(pro-2-yn-1-ylamino)hexylamine (Compound 3, Fig. 3 tert-Butyl(6-(pro-2-yn-1-ylamino)hexyl)carbamate

(514 mg, 2.0 mmol) was subjected to a solution of 10% TFA in dichloromethane (15 ml) for 3 h. The water was added and the organic phase was separated. Then the water phase was neutralized with 10% NaHCO_3_ to pH 8.0. Then removal of water is evaporated under reduced vacuum. Dichloromethane was used to wash the residue and combine organic solvent. The product was obtained through removing dichloromethane and as a yellow oil (300 mg, 96%) The crude product was used directly for next step.

#### Synthesis of Adenosine-6-amine

A mixture of 6-chloro-9-(β-D-ribofuranosyl)purine (527 mg, 1.84 mmol), compound 3 (300 mg, 1.94 mmol), 1.84 mmol of Et_3_N and 25 ml of ethanol was refluxed at 60 °C for 18 h^26^. After completion of the reaction the solvent was evaporated under high vacuum. Purification using silica gel chromatography (CH_3_OH:dichloromethane = 1:10) and the product was gained as white solid (380 mg, 50%) yield. ^1^H NMR (DMSO-d_6_) δ 8.34 (s, 1 H), 8.21 (s, 1 H), 7.9 (s, NH, 1 H), 5.89 (d, *J* = 8 Hz, 1 H), 5.44 (d, *J* = 4 Hz, 2 H), 5.19 (d, *J* = 4 Hz, 1 H), 4.63 (m, 1 H), 4.17 (d, *J* = 4 Hz, 1 H), 3.97 (t, 1 H), 3.70 (dd, J = 12 HZ, J = 4 Hz, 1 H), 3.58 (dd, J = 12 Hz, J = 4 Hz, 1 H), 3.47 (s, 2 H), 3.12 (m, 2 H) 2.51 (t, 2 H), 1.60 (m, 2 H), 1.40 (s, 2 H), 1.31 (m, 4 H) ppm. IR (Nujol): υ = 3504 (w), 3343 (s), 3264 (s), 3148 (s), 2927 (m), 2855 (s), 2150 (w), 1800 (m), 1625 cm^−1^ (s).

#### Synthesis of methyl 2,3-O-(1-methylethylidene)-5-O-2-propyn-1-yl-β-D-ribofuranoside (Compound 5, Fig. 4)

Methyl 2,3-O-isopropylidene-β-D-ribofuranoside (4; 4.0 g, 20 mmol) was dissolved in dry dimethylformamide (DMF; 30 ml)^27–30^. This was cooled (0 °C), and NaH (60% in mineral oil, 1.78 g, 23 mmol) was slowly added. The mixture was allowed to warm to room temperature and cooled again, and propargyl chloride (0.31 mol) was added very slowly. The mixture was stirred at room temperature overnight. The mixture was treated with methanol (10 ml) and concentrated in vacuum. It was coevaporated with toluene (2 × 10 ml). The (black) mixture was extracted with water and ethyl acetate (25 ml each). The water layer was subsequently extracted with CH_2_Cl_2_. The organic layers were combined, dried (MgSO_4_), and concentrated. The residue was purified by column chromatography (Eluent 10% CH_3_OH in ethyl acetate): yield 3.0 g (12.5 mmol, 62%).

#### Synthesis of 5-O-propargayl-R,β-D-ribofuranose (Compound 6, Fig. 4). methyl 2,3-O-(1-methylethylidene)-5-O-2-propyn-1-yl- β-D-Ribofuranoside

(3 g, 12.5 mmol) was dissolved in 60 ml of HCl (0.04 M) and was refluxed for 2 h^25^. The solution was neutralized with BaCO_3_, filtered, and concentrated. The mixture was purified by column chromatography (Eluent 10% MeOH in Ethyl acetate): yield 893 mg (4.7 mmol, 38%).

#### Synthesis of 5-O-propargayl-R,β-D-ribofuranose triacetate (compound 7, Fig. 4)

5-O-propargayl-R,β-D-ribofuranose (4.7 mmol) was dissolved in 50 ml of pyridine^25^. A catalytic amount of (dimethylamino)pyridine (DMAP) (60 mg, 0.47 mmol) and acetic anhydride (1.2 g, 11.75 mmol) were added. The mixture was stirred for 2 h at room temperature, concentrated in vacuum and coevaporated with toluene. The oil was extracted with water and ethyl acetate (25 ml each). The organic layer was dried (MgSO_4_), concentrated, and purified by column chromatography (Eluent: ethyl acetate): yield 1.09 g (3.5 mmol, 74%). 1 H NMR (CDCl_3_) δ 6.16 (s, 1 H), 5.43 (m, 1 H), 5.36 (m, 1 H), 4.39 (m, 1 H), 4.31 (s, 2 H), 3.72 (dd, 2 H), 2.45 (s, 1 H), 2.11 (3 × s, 9 H) ppm.

#### Synthesis of 6-chloro-9-(5-O-2-propyn-1-yl-β-D-ribofuranosyl)-9H-Purine 3′,4′-diacetate (Compound 8, Fig. 4)

Silylation of 6-Chloropurine was accomplished by treating (770 mg, 5 mmol) of the compound with 1,1,1,3,3,3-hexamethyldisilazane (HMDS; 20 ml, 93.1 mmol) and 50 µL of chlorotrimethylsilane (TMSCl; 0.4 mmol) at 130 °C for 20 h^25^. The silylated compound was concentrated and used without further purification. Vorbruggen Coupling was carried out by first adding the silylated purine (5 mmol) to *5-O-propargayl-R,β-D-ribofuranose triacetate* (3.5 mmol) in 7.5 ml of dry 1,2-dichloroethane. The resulting solution was coevaporated twice with dry 1,2-dichloroethane and subsequently dissolved in 75 ml of dry 1,2-dichloroethane. The solution was gently refluxed, and after 5 min TMS triflate (0.5 ml, 2.58 mmol) was added. The mixture was refluxed for 2 h, cooled to room temperature, and diluted with CH_2_Cl_2_. It was extracted with 5% NaHCO_3_ and water. The organic layer was dried (MgSO_4_), concentrated, and 6-Chloro-9-(3,4-di-O-acetyl-5-O-propargyl-β-D-ribofuranosyl)-purine was purified by column chromatography (eluent 3% acetone in DMC) to yield 700 mg (49%). ^1^H NMR (CDCl_3_) δ 8.80 (d, 1 H), 8.73 (d, 1 H), 5.83 (d, J = 5 Hz, 1 H), 5.62 (d, J = 5 Hz, 1 H), 5.34 (dd, J = 5 Hz, 1 H), 4.46 (s, 1 H), 4.33 (m, 2 H), 3.59 (dd, 2 H), 2.56 (s, 1 H), 2.05 (3 × s, 9 H) ppm.

#### Synthesis of 5-O-propargyl-adenosine (compound 9, Fig. 4)

Compound 8 (700 mg, 1.71 mmol) was dissolved in 4% NH_3_/EtOH (30 ml), and the mixture was stirred overnight at room temperature^26^. The mixture was concentrated and purified by column chromatography (Eluent 10% methanol in dichloromethane) to yield 300 mg (58%). ^1^H NMR (CDCl_3_) δ 8.72 (s, 1 H), 8.53 (s, 1 H), 6.16 (d, J = 5 Hz, 1 H), 4.70 (s, NH, 1 H), 4.65 (d, J = 5 Hz, 1 H), 4.50 (d, J = 5 Hz, 1 H), 4.45 (d, J = 5 Hz, 1 H), 4.18 (s, 2 H), 3.90 (d, J = 10 Hz, 1 H), 3.79 (d, J = 10 Hz, 1 H), 2.49 (s, 1 H) ppm. IR (Nujol): υ = 3258 (w), 3150 (m), 3062 (w), 2922 (s), 2852(m), 1670 (s), 1660 (m), 1582 (s), 1100 cm^−1^ (s);

#### General procedure for the reaction of acetylene-functionalized adenosine to PLA-β-PEG-N_3_

The representative synthesis (PLA-β-PEG400-N_3_) was as follows^27^. To a degassed solution of PLA-b-PEG-N_3_(200 mg, 10 µmol, 1 equiv) and alkyne adenosine (18 equiv) in anhydrous DMF (12 ml) was added with a syringe, a degassed solution of CuBr (10 mg, 69 µmol, 6.9 equiv) and PMDETA(37 µL, 0.179 mmol, 17 equiv) in anhydrous DMF(800 µL). The reaction mixture was stirred for 16 h at 65 °C under nitrogen. The solution was concentrated under reduced pressure and the residue was dissolved into chloroform, then washed with saline solution and water until the organic phase become colorless. Then the organic phase was dried by anhydrous sodium sulfate and removed by rotary evaporation. The residue was dissolved in minimum amount of THF, and further precipitate in water for third times. The precipitate was freeze-dried to yield a white powder.

#### General procedure for nanoparticle preparation

The copolymer attached with adenosine (30 mg in total) was dissolved in ethyl acetate (1.2 ml)^26^. The above organic phase was added to 3.3 ml of an aqueous phase containing 1% w/v Pluronic F68. The mixture was then vigorously shaken using a vortex shaker for 1 min. The resulting emulsion was ultrasonicated (using an ultrasonic probe) for 3 min and the organic solvent was removed under reduced pressure using a rotary evaporator. The resulting nanoparticle suspension was ultracentrifuged at 1600 g for 45 min and the pellet was resuspended in 3 ml of pH = 7.4 PBS. The nanoparticles were filtered through a 1 µm glass filter fist and then 0.45 µm and stored at 4 °C until use.

### Characterization of nanoparticles

#### NMR spectroscopy

NMR spectroscopy was performed in CDCl_3_ or DMSO-*d*_6_. ^1^H NMR spectroscopy was performed on a Bruker Avance 500 spectrometer at 500 MHz(^1^H).

#### Concentration of nanoparticles

**Figure 10:**
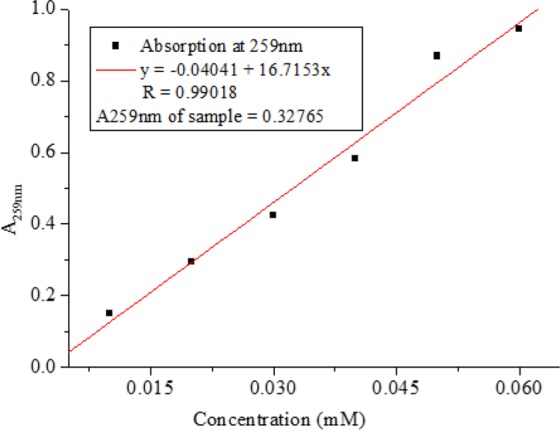
The best-fit line of the standard solution of adenosine.

Based on the Beer’s Law, the various standard solution of adenosine is made and being quantified through calorimetric assays. Then the data was graphed based on the concentrations and a Best-Fit line was gained through the points (Fig. 10). From the slope of the best–fit line together with the absorbance, the concentration of nanoparticles can be calculated.

#### Dynamic Light Scattering (DLS)

Measurement of the nanoparticle diameter was performed using a Nano ZS from Malvern (90° scattering angle) at 20 °C. The particle size distribution values are given by the DLS apparatus. DLS was run for every new batch of nanoparticles. The sample was diluted until a good PDI value was obtained, showing a narrow size distribution of the nanoparticles.

#### Transmission Electron Microscopy (TEM)

Place 3 µL of sample onto carbon coated 400 mesh Cu/Rh grid (Ted Pella Inc, Redding, CA) and stain with 1% uranyl acetate in distill water (polysciences, Inc, Warrington, PA). Stained grids were examined under FEI Talos120C transmission electron microscope and photographed with a Gatan OneView digital camera.

#### Measurement of cyclic AMP in macrophage cell line

RAW264.7 cells were plated in 12 well/plate (500,000 cell/well). The cells were pre-treated for 10 minutes at 37 °C with 4-(3-butoxy- 4-methoxybenzyl)-2-imidazolidinone in order to inhibit phosphodiesterases. Cells were then treated with adenosine (1 µM) and nanoparticles (1 µM) for 10 minutes. A2BR and A2AR antagonists, respectively PSB1115 (1 µM) and ZM241385 (1 µM) were added at the last 5 minutes of the previous incubation. After incubation cells were washed with cold PBS and incubated with 0.1 M of HCl for 10 minutes at RT. Cytoplasmic cell content was collected and centrifuged at 600 g. cAMP amount was evaluated by using a kit following the manufacture instructions (Enzo Life science).

#### Mouse primary chondrocyte extraction and culture

Articular mouse chondrocytes were obtained from C57BL/6 mice. After sacrifice of 5/7-day-old mice, the femurs were dislocated from the hip. Soft tissue was removed and the femoral head, femoral condyles, and tibial plateau were isolated and placed in culture medium (DMEM supplemented with 2 mM-glutamine, 50 U/mL penicillin, and 0.05 mg/mL streptomycin). Cartilage tissue was incubated in a solution of collagenase D (3 mg/ml) for 45 minutes at 37 °C. After agitation, the tissue was incubated in a new collagenase D solution under the conditions previously described. Cartilage pieces were then incubated overnight at 37 °C in a new solution of collagenase D (0.5 mg/ml). Sheets of cells were detached from the bone, passed through a 72-µm nylon mesh and plated at a density of 8 × 10^3^ cells/cm^2^.

Primary chondrocytes (80% confluence) were starved overnight and treated for 24 h with mouse recombinant IL-1β (5 ng/ml). For the evaluation of NF-kB nuclear translocation, cells were collected and nuclear and cytosolic protein components were separated using NE-PER kit (Thermo Scientific) following the manufacturer’s protocol.

Total and nuclear protein fractions were quantified using the BCA kit (Thermo Scientific). Western blotting was performed loading 4 µg of protein. Proteins were separated by electrophoresis through 10% polyacrylamide gels by SDS-PAGE and then transferred to a nitrocellulose membrane. Non-specific antibody binding was blocked with 3% BSA in TBS/Tween 20 buffer. Membranes were incubated overnight at 4 °C with the specific primary antibody (1:1000), and after washing, incubated with goat anti-rabbit IRDye 800 CW and goat anti-mouse IRDye 680 RD (1:5000). Membranes were scanned with Li-cor Odyssey equipment and the intensity of the protein bands were quantified by densitometric analysis using Image Studio 2.0.38 software.

#### Reverse transcription and Real Time PCR

RNA extraction was performed from mouse primary chondrocytes using RNeasy Mini Kit (Qiagen, Invitrogen) and QIAshredder colums (Qiagen, Invitrogen), following the manufacturer’s protocol. RNA reverse transcription was performed using the MuLV Reverse Transcriptase PCR Kit (Applied Biosystems). After RNA reverse transcription to cDNA, real time PCR reactions were performed for a relative quantification of collagen X, MMP-13 and IL-6 performed on a Stratagene Mx3005P (Agilent Technologies, CA, USA) with Brilliant SYBR Green Kit QPCR Master Mix (Stratagene, Agilent Technologies, CA, USA), according to the manufacturer’s protocol.

#### MTT assay

Cell viability was performed in RAW 164.7 cell line after 24 hours treatment with the compound PLA-PEG2000-3,4-OH-Adenosine (1 µM) or adenosine alone (1 µM). The assay was performed following the protocol provided by the manufacture (CellTiter 96^®^ Non-Radioactive Cell Proliferation Assay-Promega, CA, USA).

#### Induction of post-traumatic OA (PTOA) in rats and treatment with nanoparticles

The posttraumatic OA (PTOA) model used is a non-invasive method for inducing anterior cruciate ligament (ACL) rupture in rat knees *in vivo* with a single load of tibial compression. We chose this model in rats because of the technical ease in injecting the knees of these animals and because correction of an induced lesion would better mimic the situation in humans. The procedure was performed under anesthesia (1–3% isoflurane) as previously described^12^. All experimental groups of rats, as described below, consisted of 3 rats and each experimental group was repeated once (total of 6 rats per experimental group). This number of rats was chosen because larger group sizes led to operator overload and diminished quality of results. Animals were not randomized for these studies.

Rats were treated with intra-articular injections of 100 ml of a liposomal suspension containing a high concentration of adenosine (10 mg kg/1), empty liposomes or with saline for 8 weeks. The animals were separated into two main cohorts, the prevention group received injections commencing immediately after ACL rupture, and the treatment group received the first injection 7 days after ACL rupture. Injections were performed every 10 days thereafter in both groups. Knee swelling and weight in the rats were measured before every injection. At the end of the experiment rats were killed and both legs were collected for immunohistochemistry and microcomputed tomography (μCT) analysis^12^.

#### µCT Cartilage examination

After sacrifice both legs were excised and fixed with 4% PFA for 48 hours and then preserved in 70% ethanol. After washing with PBS, rat knees (femoral and tibial surfaces, n = 3 for each group) were incubated in PBS containing the ionic contrast agent Hexabrixr (40% v/v) for 6 hours. All joints were evaluated in a (16 mm) scanning tube providing a volex size of 10.5 µm and scanned at 55 kV, 181 µA, and 110 minutes of acquisition time. During scanning the samples were wrapped in paper soaked in PBS to avoid dehydration^12^.

After µCT analysis the samples were washed with PBS and decalcified in 10% EDTA for 4 weeks. Paraffin-embedded histological sections (5 µm) were cut, mounted and prepared for analysis with H&E and Safranin O/Fast green staining to assay different cartilage components. Slides were scanned using a Leica microscope equipped with Slidepath Digital Image Hub version 3.0 Software.

#### Data analysis

µCT analysis of bone and cartilage volume was performed using CTvox software to reconstruct 2D and 3D images and to calculate various bone characteristics. Amira software (FEI, Oregon-USA) was used to reconstruct mice and rat joints from µCT data based on differential density of bone and Hexabrix-treated cartilage. Statistical significance for differences between groups was determined using Student’s T-test, two-way or one-way ANOVA, as appropriate, using GraphPad software (GraphPad, San Diego, CA). If the overall differences were significant (F < 0.05) then differences between groups were analyzed by Bonferroni post-hoc testing.

#### OARSI Score

Histology and immunohistochemistry were performed as previously described^27^ and OARSI score determined blindly for each specimen taking into account the severity of cartilage degradation, cartilage calcification, presence of osteophytes and their size.

### Ethical Approval and Informed Consent

All experiments with animals were reviewed and approved by the NYU School of Medicine Institutional Animal Care and Use Committee.

## Supplementary information

Supplementary material

## Data Availability

The data that support the findings of this study are available from the corresponding author on request.
