# Supplementary material for: Adenosine-Functionalized Biodegradable PLA-b-PEG Nanoparticles Ameliorate Osteoarthritis in Rats
Source: Sci Rep. 2019 May 15;9:7430. doi: 10.1038/s41598-019-43834-y (PMC6520388; doi:10.1038/s41598-019-43834-y)

Adenosine-Functionalized Biodegradable PLA-b-PEG Nanoparticles Ameliorate Osteoarthritis in Rats

Xiuling Liu,[1] Carmen Corciulo,[2] Stephanie Arabagian,[1] Abraham Ulman,*[1] Bruce N. Cronstein*[2]

[1]Department of Chemical and Biomolecular Science and Engineering, NYU Tandon School of Engineering, 6 Metrotech Center, Brooklyn, NY 11201. [2]Department of Medicine, NYU School of Medicine, 550 First Avenue, New York, NY 10016

*Corresponding authors. Email: au323@nyu.edu, Bruce.Cronstein@nyumc.org

Supplementary Material

Synthesis of PEG-N₃.[18] PEG400 and PEG2000 had been tried as the hydrophilic spacers connecting the adenosine to the PLA nanoparticles. Since nanoparticles where PEG2000 had been used showed bioactivity, we only discuss the corresponding synthetic procedures. The preparation of azide-terminated PEG required two steps. In the first the mono para-toluene sulfonyl ester (tosylate) was prepared, by reacting excess PEG with para toluene sulfonyl chloride using 4-dimethylaminopyridine (DMAP) as the base. The product was precipitate from dichloromethane and diethyl ether.

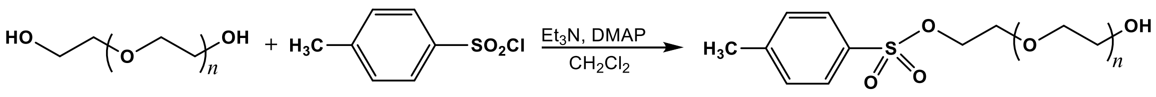

The monotosylate was reacted with sodium azide (NaN_3) in DMF to provide the azide-terminated PEG.[18]

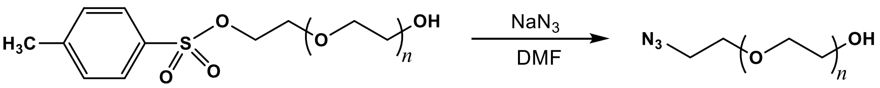

Synthesis procedure of α-tosyl-ω-hydroxyl PEG.[18] PEG, previously dried by azeotropic distillation in toluene using a Dean-Stark trap, was dissolved in 250 ml dry toluene, and Ag_2O (1.5 equiv, 4.8 g, 20.7 mmol) and KI (0.2 equiv, 458 mg, 2.76 mmol) were added. To this rapidly stirred solution p-toluene sulfonyl chloride (1.05 equiv, 2.76 g, 14.5 mmol) was added in one portion. The reaction mixture was left at room temperature with constant stirring for 12 h before filtration over a filter cell cake. Solvent removal by rotary evaporation was performed. The crude product was dissolved in 20 ml dichloromethane and then precipitated by dropwise addition into diethyl ether, the polymer was collected by filtration (98%).

Synthesis of α–azide-ω-hydroxyl PEG.[18] α-Tosyl-ω-hydroxyl PEG (10 g, 6.23 mmol) and NaN_3 (2 g, 31 mmol) were dissolved in 150 ml of dry DMF and the mixture was stirred at 90°C under

nitrogen for overnight. Then the DMF was removed under reduced vacuum. The crude product was dissolved in 100 ml dichloromethane and washed twice with brine and twice with water. The Organic layer was dried over sodium sulfate, reduced to small volume by rotary evaporation and finally precipitated by dropping into diethyl ether. The polymer was collected by filtration.

Western blot described in figure 8

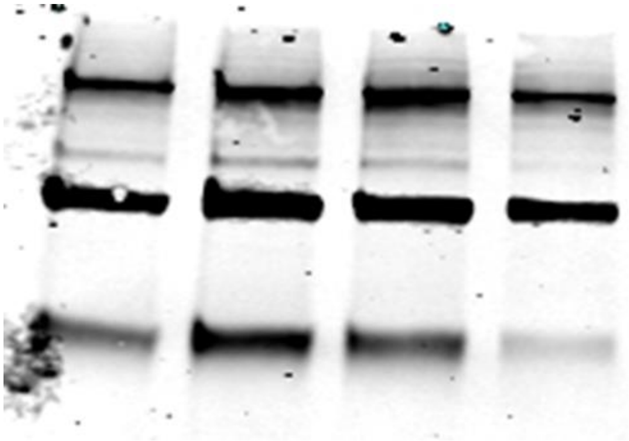

Supplement: Supplementary file 1 — Supplementary material [file 41598_2019_43834_MOESM1_ESM.pdf]
